# Disentangling Hippocampal and Amygdala Contribution to Human Anxiety-Like Behavior

**DOI:** 10.1523/JNEUROSCI.0412-19.2019

**Published:** 2019-10-23

**Authors:** Dominik R. Bach, Martina Hoffmann, Carsten Finke, Rene Hurlemann, Christoph J. Ploner

**Affiliations:** ^1^Computational Psychiatry Research, Department of Psychiatry, Psychotherapy, and Psychosomatics, University of Zurich, CH-8032 Zurich, Switzerland,; ^2^Wellcome Centre for Human Neuroimaging and Max Planck/UCL Centre for Computational Psychiatry and Ageing Research, University College London, London WC1N 3BG United Kingdom,; ^3^Department of Neurology, Charité–Universitätsmedizin Berlin 10117 Berlin, Germany,; ^4^Berlin School of Mind and Brain, Humboldt-Universität zu Berlin, 10117 Berlin, Germany,; ^5^Department of Psychiatry and Division of Medical Psychology, University of Bonn, 53127 Bonn, Germany, and; ^6^Department of Psychiatry, University of Oldenburg Medical Campus, 26160 Bad Zwischenahn, Germany

**Keywords:** anxiety-like behavior, approach decision, approach–avoidance conflict, clinical lesion models, double-dissociation, escape vigor

## Abstract

Anxiety comprises a suite of behaviors to deal with potential threat and is often modeled in approach–avoidance conflict tasks. Collectively, these tests constitute a predominant preclinical model of anxiety disorder. A body of evidence suggests that both ventral hippocampus and amygdala lesions impair anxiety-like behavior, but the relative contribution of these two structures is unclear. A possible reason is that approach–avoidance conflict tasks involve a series of decisions and actions, which may be controlled by distinct neural mechanisms that are difficult to disentangle from behavioral readouts. Here, we capitalize on a human approach–avoidance conflict test, implemented as computer game, that separately measures several action components. We investigate three patients of both sexes with unspecific unilateral medial temporal lobe (MTL) damage, one male with selective bilateral hippocampal (HC), and one female with selective bilateral amygdala lesions, and compare them to matched controls. MTL and selective HC lesions, but not selective amygdala lesions, increased approach decision when possible loss was high. In contrast, MTL and selective amygdala lesions, but not selective HC lesions, increased return latency. Additionally, selective HC and selective amygdala lesions reduced approach latency. In a task targeted at revealing subjective assumptions about the structure of the computer game, MTL and selective HC lesions impacted on reaction time generation but not on the subjective task structure. We conclude that deciding to approach reward under threat relies on hippocampus but not amygdala, whereas vigor of returning to safety depends on amygdala but not on hippocampus.

**SIGNIFICANCE STATEMENT** Approach–avoidance conflict tests are widely investigated in rodents, and increasingly in humans, to understand the neural basis of anxiety-like behavior. However, the contribution of the most relevant brain regions, ventral hippocampus and amygdala, is incompletely understood. We use a human computerized test that separates different action components and find that hippocampus, but not amygdala, lesions impair approach decisions, whereas amygdala, but not hippocampus, lesions impair the vigor of return to safety.

## Introduction

Appropriate behavior in the face of conflicting goals is key to arbitrating many biological scenarios, and it is particularly challenging when threat is involved such as during foraging and exploration under predation. A laboratory model of this situation is provided by approach–avoidance conflict tests (Calhoon and Tye, 2015), often regarded as reflecting aspects of clinical anxiety in humans (Gray and McNaughton, 2000; Calhoon and Tye, 2015; Bach et al., 2018). A body of literature demonstrate that the ventral (in rodents) or anterior (in humans) hippocampus (HC) is involved in behavioral control in such tests (for comprehensive reviews, see Gray and McNaughton, 2000; Ito and Lee, 2016; Kirlic et al., 2017), and somewhat less consistently, the amygdala (Kirlic et al., 2017). In humans, we have previously shown that degenerative HC (Bach et al., 2014) and amygdala lesions (Korn et al., 2017) impact on an anxiolytic-sensitive (Korn et al., 2017; Bach et al., 2018) approach–avoidance conflict test. Yet, the mechanistic function of these areas remains debated (Ito and Lee, 2016). Beyond a well known role of the HC for spatial cognition and memory, several suggestions for its function in approach–avoidance conflict have been put forward: that ventral HC is involved in behavioral inhibition when conflict is detected (Bannerman et al., 2014); that it represents threat aspects of the situation, the removal of which would reduce threat-related behavior (Gray and McNaughton, 2000); and/or that it inhibits representation of reward aspects (Ito and Lee, 2016) with possibly distinct roles for HC subfields (Schumacher et al., 2018).

Notably, many classic approach–avoidance conflict tests share the limitation that their behavioral readouts collapse several distinct actions. This is particularly the case for ethological tests such as elevated plus maze (Pellow et al., 1985) or open-field test (Montgomery, 1955), which combine several components of approach behavior as well as withdrawal from danger, and decision processes, into a small number of readouts (Rodgers et al., 1997). Some of these actions, such as active avoidance or escape, are not known to require hippocampus in non-conflict situations (LeDoux et al., 2017; Evans et al., 2018), but a direct comparison is difficult. Our previously proposed human approach–avoidance test (Bach et al., 2014), a computerized translation of open field test, is imbued with the same problems.

In contrast, operant conflict tests in rodents (Geller and Seifter, 1960; Vogel et al., 1971) and nonhuman primates (Chudasama et al., 2008; Amemori et al., 2015) in principle allow separating action components (for an example in mice: Oberrauch et al., 2019). Here, we capitalize on a human operant conflict test (Bach, 2015), which measures a decision to approach (action), the vigor with which this action is implemented (approach latency), and the vigor of the retreat to safety (return latency). All these behavioral components are influenced by the probability of virtual predation (threat level) and the possible loss involved: healthy humans reduce approach, delay approach, and accelerate return, when the situation is more dangerous (Bach, 2015). While reducing approach and accelerating return is reward maximizing, delaying approach is not. However, it reminisces novelty-suppressed feeding (Britton and Britton, 1981), another rodent approach–avoidance test, and can be explained under particular subjective assumptions about the task parameters (Bach, 2015, 2017). Furthermore, we have previously shown with magnetoencephalography that hippocampus may be involved in behavior in this task (Khemka et al., 2017).

Here, we investigated three patients with unilateral medial temporal lobe (MTL) lesions, one patient with selective bilateral HC damage, and one patient with selective bilateral amygdala lesion. We hypothesized that hippocampus, but not amygdala, lesions impact on the decision to approach. In our previous approach–avoidance conflict test, behavior was particularly impaired by HC lesions and anxiolytics when potential loss was high (Bach et al., 2014, 2018; Korn et al., 2017), such that we expected here a lesion × potential loss interaction. Based on the amygdala's role in non-conflict active avoidance (LeDoux et al., 2017), we also hypothesized that amygdala but not hippocampus lesions impact on return to safety under conflict.

## Materials and Methods

### 

#### Participants

We recruited three patients with postsurgical unilateral MTL lesions affecting hippocampus, amygdala, and adjacent neocortex, together with 10 control participants; one patient with highly selective bilateral HC lesions, and 9 control participants; as well as one patient with bilateral selective amygdala lesions due to Urbach–Wiethe syndrome, together with 26 controls (Table 1). All controls were age- and sex-matched.

**Table 1.** Patient and lesion characteristics

Patient	Age, y	Sex	Lesion										Etiology	Clinical note
			Right MTL					Left MTL						
			AMY	HC	ERC	PRC	PHC	AMY	HC	ERC	PRC	PHC		
MTL 1	51	F	+	+	+	+	−	−	−	−	−	−	Pilomyxoid astrocytoma, symptoms <1 year before resection, testing 133 months after surgery	No relapse, seizure free, visuospatial memory deficits
MTL 2	32	M	+	+	++	++	−	−	−	−	−	−	Neuroepithelial tumor, symptoms 1 year before resection, testing 107 months after surgery	No relapse, seizure free, visuospatial memory deficits
MTL 3	41	F	+	+	+	++	−	−	−	−	−	−	Epidermoid tumor, symptoms 3 years before resection, testing 152 months after surgery	No relapse, seizure free, visuospatial memory deficits
HC	26	M	−	+++	−	−	−	−	+++	−	−	−	Autoimmune encephalitis, onset 10 days before testing	Severe amnesic syndrome
Amy	43	F	+	(+)	−	−	−	+	(+)	−	−	−	Congenital Urbach–Wiethe syndrome (*de novo* mutation), testing 31 years after first symptoms	Seizure free, social and affective deficits

AMY, amygdala; ERC, entorhinal cortex; PRC, perirhinal cortex; PHC, parahippocampal cortex; +, indicates a rostrocaudal lesion extent of ≤20 mm; ++, ≤40 mm; +++ >40 mm; (+), indicates involvement of the amygdalo-hippocampal border; −, indicates an unaffected region.

Postsurgical MTL lesions resulted from resection of benign brain tumors in all three patients and always affected the right amygdala, anterior hippocampus, entorhinal cortex, and parts of perirhinal cortex. The parahippocampal cortex was spared in all patients. Onset of presurgical symptoms was during adulthood. All patients had already participated in previous investigations of our group (for clinical details, imaging, and neuropsychological findings, see Braun et al., 2008; Finke et al., 2008; Esfahani-Bayerl et al., 2016). Patients suffered from mild visuospatial memory deficits but were fully independent in daily life activities.

In the HC patient, exceptional selective bilateral HC lesions resulted from autoimmune encephalitis. In this patient, both hippocampi were equally affected across the entire rostrocaudal extent. Entorhinal cortex, perirhinal cortex and parahippocampal cortex were completely spared (for clinical details and neuropsychological findings, see Esfahani-Bayerl et al., 2019). Similar to the cases reported by Rempel-Clower et al. (1996), this patient suffered from a retrograde and anterograde amnesic syndrome that severely affected autobiographical events, verbal, visual and spatial memory, whereas some other memory domains were spared or less affected (e.g., memory for music and faces).

MTL and HC patients were tested in 2016 at Charité University Hospital in Berlin.

In the amygdala patient (previously labeled B.G., or Patient 2; Becker et al., 2012), lesions encompassed most of bilateral amygdalae while both hippocampi were almost unaffected. Neuropsychology and imaging findings for B.G. have been extensively covered in previous reports (Talmi et al., 2010; Bach et al., 2011, 2013, 2015; Becker et al., 2012; Korn et al., 2017). The patient is impaired in anterograde and retrograde interference of emotional pictures on memory (Hurlemann et al., 2007), phonemic fluency and short-term concentration (Talmi et al., 2010), free verbal recognition of fearful faces, startle potentiation by threat-related scenes, social network size (Becker et al., 2012), and prioritization of angry over happy face expression (Bach et al., 2015). The patient was tested in 2017 at age 43 at the University Hospital in Bonn; her twin sister was not tested.

All control participants were distinct from those previous studies using the same or similar setups (Bach, 2015, 2017; Khemka et al., 2017). From the amygdala control group, we excluded two participants because of low performance (i.e., low number of trials in which they approached the token and survived the virtual predator): their performance was >4 SD below the mean of the rest of this control group, ∼2 SD below the next worst performing participant of the rest of the control group, and smaller than any patient or control participant in the MTL/HC sample. The study was in full accordance with the Declaration of Helsinki and approved by the respective local research ethics committees.

#### Design and procedure: approach–avoidance conflict task

**Figure 1.**
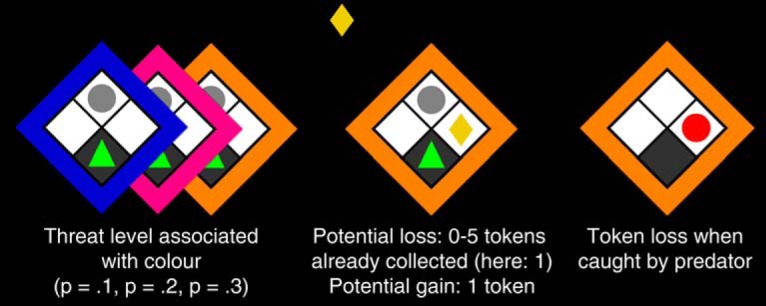
Behavioral task. On each trial, a human player (green triangle) rests in a safe place on the bottom of grid, while a “predator” is sleeping at the top (gray circle). On each epoch, up to six successive reward tokens (yellow rhombi) appear. To obtain a token, the player uses the left/right cursor keys to move out of the safe place and back. The colored frame indicates the threat level of the sleeping predator with color/threat association balanced across subjects. When caught, all tokens are lost. Potential loss is the number of tokens already collected on this epoch. (*p*: probability to get caught per 100 ms outside of safe place).

This operant conflict test kept approach incentive constant and varied avoidance incentives in a 3 × 6 factorial design with the within-subjects factors “threat level” (wake-up probability of the virtual predator: low/medium/high) and “possible loss” (0–5 tokens). Participants played 4 (MTL/HC lesion/controls) or 6 (amygdala lesion/controls) blocks of 45 successive epochs of a previously published computer game (Bach, 2015) on a 2 × 2 grid, presented with ∼4° vertical visual angle on a standard LCD monitor (Fig. 1). To allow direct comparison between MTL/HC and amygdala lesions, with different numbers of task blocks, only the first four blocks were included in the analysis. The human player was controlled with the left/right cursor keys on a standard computer keyboard and could move between the lower three grid blocks any time unless caught by the predator. Each move between adjacent grid blocks required a single key press. The player started each epoch in the “safe” bottom grid block. In each epoch, a sequence of up to six reward tokens appeared at random time points in a random (left/right) location. The player could decide each time whether or not to approach and collect the token by moving to its location. Participants received a fixed payment and an additional reward for the number of retained tokens of one randomly drawn epoch at the end of the experiment. A “sleeping predator” was waiting above the token and could become active if the human player was outside the safe place, with a probability per time unit that was constant over time (*p*_1_ = 0.1, *p*_2_ = 0.2, *p*_3_ = 0.3, for the 3 predators per 100 ms). Actual catch rates depend on participants' return latencies (see Fig. 3). It would then “eat” the human player, and all previously collected reward tokens from this epoch were removed. Once the predator was active, the human player had no possibility to escape. After catching the player, the active predator stayed visible on the screen for the remaining time of the epoch while the human player had to wait. The token stayed on the screen and could be collected for a random interval drawn from an exponential distribution with a mean of 1.25 s. If the player did not collect the token, then the token disappeared after this interval. After the predetermined disappearance time, a waiting interval with random duration started (drawn from the same exponential distribution plus 500 ms), before the next token came on the screen or the epoch ended.

#### Design and procedure: safe predator exposure Task 2 (MTL/HC sample)

In Blocks 5–6, participants in the MTL/HC sample were given a different task on 36 epochs per block, randomly interspersed with 9 epochs of approach–avoidance conflict Task 1. Task 1 epochs from these blocks were not included into the analysis to allow direct comparison with the amygdala lesion patient. The type of task was graphically signaled by a gray rhombus (approach–avoidance task) or a gray circle (safe predator exposure task) below the grid. The graphical setup of Task 2 was exactly the same as in Task 1, but participants could not move on the grid and always stayed in the safe place. They were asked to “expose” the awake predator by pressing the cursor up key. If the predator was awake at this point in time, it would turn red, and the next epoch would start. If the predator was sleeping, it would turn black for 100 ms and the epoch would continue. This feedback gave participants an opportunity to learn the experimental statistics, according to which the probability of being awake was independent of time, or of token appearance. On each epoch, the human player had six attempts to expose the predator, after which the key was disabled until the epoch ended. Participants were explicitly informed that the tokens could not be collected. The duration of the task depended on participants' behavior: they could shorten the task by attempting to expose the predator independently from the tokens. One randomly selected epoch from Task 2 was rewarded at the end of the experiment; if the participant successfully exposed the predator, they gained as much as from collecting two tokens in Task 1. The objective wake-up probabilities of the three predators for each exposure attempt were *p*_1_ = 0.1, *p*_2_ = 0.2, *p*_3_ = 0.3, and constant over time.

#### Design and procedure: memory test

After the last trial, participants were asked, for each of the three threat levels, to indicate how likely it was that they got caught if they left the safe place. Participants were shown the grid with the frame color, and asked to make a rating on a visual analog scale anchored with “0%” and “100%” (see Fig. 3).

#### Data analysis

All inference statistics were computed in the software R (https://www.r-project.org). We first tested the entire control group's behavior in the task to ensure consistency with previous publications. We then compared the MTL lesion with its control group in linear mixed effects (LME) model to identify potential consequences of amygdala and/or hippocampus lesions. We then extracted respective coefficients and compared the effect of MTL lesions with the effect of selective hippocampus or amygdala lesions, to clarify the contribution of amygdala and hippocampus. Single-trial data for approach and return latency are necessarily unbalanced because the number of data points for each cell in the design depends on behavioral choices and on chance. This is why LME models are more appropriate than a traditional ANOVA approach.

##### Decision to approach.

Decision to approach was reconstructed by creating six data points for each epoch, corresponding to the possibility of collecting six tokens. For each of these six tokens, we scored 0 if the individual chose to collect less than this number of tokens and 1 otherwise. Choices in epochs on which the player was caught cannot be reconstructed and were therefore not analyzed. The resulting single-trial data are serially correlated by design. To reduce this correlation, they were averaged within conditions, and we analyzed the proportion of approach responses in a [2 (group) ×] 3 (threat level) × 6 (potential loss) LME model with random subject intercept. Fixed-effects *F* tests were based on un-partitioned error variance and Satterthwaite approximation to degrees of freedom, which appropriately controls the false-positive rate (Luke, 2017; using the R functions ANOVA and lmerTest). We then applied Greenhouse–Geisser correction for violations of multisphericity.

##### Approach and return latency.

For each trial on which participant approached the token, we extracted the approach latency, and if the player was not caught, also the return latency. To avoid response latencies being biased by extreme values, they were only analyzed if they fell into response windows of 150 ms < approach latency < 2000 ms and 0 ms < return latency < 2000 ms, as in previous work (Bach, 2015, 2017). Most players rarely collected the sixth token such that some design cells were empty and the parameters could not be estimated reliably. Therefore, the sixth token was excluded for all RT analysis. RT data on the single-trial level were analyzed in a [2 (group) ×] 3 (threat level) × 5 (potential loss) LME model. We did not transform reaction times, because we had no a priori reason to do so, and a previous report demonstrated that analysis of log-transformed reaction times replicates analysis of raw RTs (Bach, 2015).

##### Comparison between patients.

To compare selective and unselective lesions, we used an ordinal approach based on summary statistics, to avoid making strong distributional assumptions on the single-case level. We computed the single-subject summary statistic reflecting the group-level significant fixed effect in the LME (linear coefficient or overall mean). We then computed the percentage rank of each patient within their respective control group. To compare one patient against a group of other patients, we used Crawford's approach for dissociation (Crawford et al., 1998; Crawford and Garthwaite, 2005a,b). This tests a null hypothesis that the difference between two test scores (here relating to 2 patients) is drawn from the same distribution as the differences between pairs of control participants. In contrast to a purely descriptive approach, it allows inferential statements whether two patients' positions in a population distribution are different (Crawford et al., 2003). We modified this approach to ordinal level, and allowed each patient to have its own control group. Thus, we created an ordinal bootstrapping test that compared the rank difference between two groups of patients with the rank differences observed in 10,000 simulations of two control groups of the same sizes as empirically used.

##### Accounting for memory impairment and other confounds.

Because of the known role of HC for declarative memory, we note that any findings relating to threat level can potentially be explained by impairment to learn the color-threat level association and do not directly speak to anxiety behavior, different from findings relating to potential loss or overall group differences. We tested the impact of MTL lesions on subjective catch rate in a group (lesion/control) × threat level ANOVA, and in a group (lesion/control) × true catch rate LME model. To account for potential differences in the overall subjective threat level (i.e., averaged across the three threat levels), we added this as a covariate (crossed with within-subject factors) to LME models with significant findings. Finally, we repeated all LMEs after adding potential confounds together with the group factor (crossed with within-subjects factors) as a covariate, namely years of education, visual memory (Rey–Osterrieth complex figure test: copy, immediate recall, delayed recall; Shin et al., 2006), and estimation of overall catch rate in the approach–avoidance task.

##### Safe predator exposure task.

We sought to determine whether participants' responses depended on the appearance of irrelevant tokens. To this end, we split the data into key presses made before the first token appeared, and those made later. For key presses after the first token, we computed the latency of each response with respect to the last token that preceded it, and analyzed the ensuing RT distributions. The distribution of these responses was compared against two null distributions that test the null hypotheses that key presses are unrelated to tokens with Kolmogoroff–Smirnoff (KS) tests. For details on the derivation of these null distributions, see Bach (2017). Differences between patients and controls were tested in a two-sample KS test, and a two-sample *t* test on mean RT per participant.

To assess the most likely source of a RT difference between patients and controls, we fit a previously derived reaction time model of the following form:

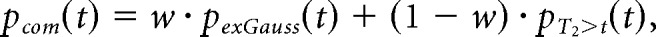
 where *p*_*T*_2_>*t*_(*t*) is the null distribution and

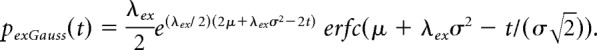
 Here, *T*_2_ is the time point of the key press with respect to the last appearing token, and λ, μ, σ, or *w* are group parameters. We compared an implementation of this model with parameters shared between patients and control participants, and implementations with group-specific parameters for λ, μ, σ, or *w*. Model parameters and likelihood were estimated using the built-in MATLAB function mle.m. We quantified model evidence as log Bayes factors (LBFs) based on Bayesian information criterion (Raftery, 1995) [LBF = 0.5 × (BIC_ref_ − BIC] and considered an absolute LBF difference >3 as decisive, in analogy to classical *p* values (Burnham and Anderson, 2004; Penny et al., 2004).

## Results

### Healthy control participants' behavior is similar to previous reports

**Figure 2.**
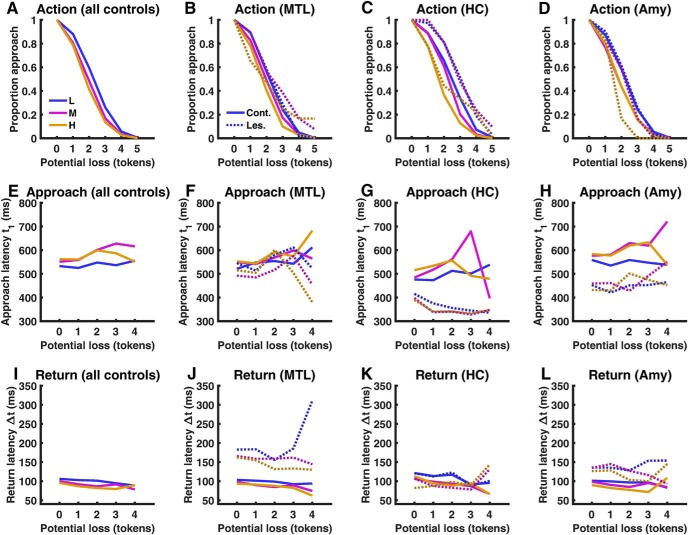
Behavioral results, displayed across the entire control group (***A***, ***E***, ***I***) and for each lesion type separately together with their respective control participants (***B***, ***F***, ***J***: MTL lesion; ***C***, ***G***, ***K***: HC lesion; ***D***, ***H***, ***L***: Amygdala lesion). Blue, Low threat level (L); purple, medium threat level (M); orange, high threat level (H). Solid lines, Control participants; dashed lines, patients. MTL, Surgical medial temporal lobe lesion; HC, selective bilateral hippocampus lesion; Amy, selective bilateral amygdala lesion. Approach and return are not displayed for the sixth token (potential loss 5 tokens) as this was rarely collected and therefore not included into statistical analysis.

We first ensured that behavior of control participants was comparable to previous reports (Bach, 2015, 2017; Khemka et al., 2017). In particular, action, approach latency, and return latency, all depended on threat level and potential loss in a linear manner (Fig. 2*A*,*E*,*J*; Table 2).

**Table 2.** LME model statistics from the combined control group (*n* = 43)

	Action (proportion approach)				Approach latency			Return latency		
	*F*	df	Epsilon	*p*	*F*	df	*p*	*F*	df	*p*
Threat level	32.48	2,714	0.9828	< 0.001	42.06	2,20,130.2	< 0.001	23.75	2,16,705.9	<0.001
Threat level: linear	62.17	1,714		<0.001	46.07	1,20,130.2	<0.001	35.11	1,16,705.9	<0.001
Potential loss	1994.63	4,714	0.311	<0.001	5.57	4,20,130.2	<0.001	23.38	4,16,705.9	<0.001
Potential loss: linear	9486.32	1,714		<0.001	11.3	1,20,130.2	0.001	31.11	1,16,705.9	<0.001
Interaction threat level × potential loss	5.86	8,714	0.2047	0.006	2.42	8,20,130.2	0.013	1.34	8,16,705.9	0.22
Interaction linear × linear	1.85	1,714		0.17	6.46	1,20,130.2	0.011	0.1	1,16,705.9	0.76

For all outcome measures, the table shows Satterthwaite approximation to degrees of freedom. For action, *p* values are based on further Greenhouse–Geisser correction to degrees of freedom using the epsilon value shown.

### Recollection of threat memory

**Figure 3.**
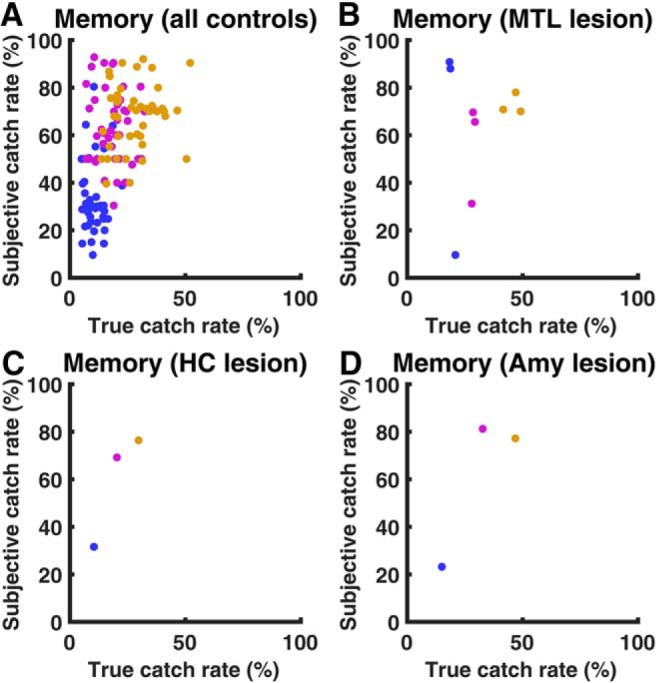
Explicit memory of catch rate after the experiment. True catch rate depends on behavior and may change over the course of the experiment. For lesion patients, color/threat association was randomized, and each control participant was presented with the same association as their respective patient. Blue, Low threat level; purple, medium threat level; orange, high threat level. ***A***: all control participants. ***B-D***: lesion patients.

The association between color and threat level depended on behavior and had to be implicitly learned during the test. The control group learned this association successfully although not precisely: ratings of catch probability strongly depended on threat level (ANOVA: *F*_(2,84)_ = 68.6; *p* < 0.001) and on true catch rate (LME: *F*_(1,127)_ = 38.4; *p* < 0.001; Fig. 3*A*). The relation between variations in true and estimated catch rate was close to perfect (regression coefficient *b* = 0.99), but there was a significant intercept (*F*_(1,127)_ = 115.8; *p* < 0.001): participants estimated catch rate 36.3% higher than the true catch rate. Comparing MTL lesion patients with their control group in an ANOVA with threat level as within-subjects factor, patients rated the catch probabilities as higher than the control group (63.7 vs 51.4%, *F*_(1,11)_ = 5.0; *p* = 0.046). However, this was largely explained by higher true catch rates in patients. In a LME model accounting for true catch rates, there was no difference between the two groups for the intercept (*F*_(1,35)_ = 1.2; *p* = 0.28) or the regression coefficient (*F*_(1,35)_ = 1.2; *p* = 0.28). Nevertheless, because of the known role of HC in declarative memory, we focused in our analysis on overall group differences, and on the impact of potential loss, rather than the impact of threat level, on behavior. Furthermore, we controlled for overall subjective catch rate as a covariate in further analyses.

### HC but not amygdala lesions impact approach decision

**Figure 4.**
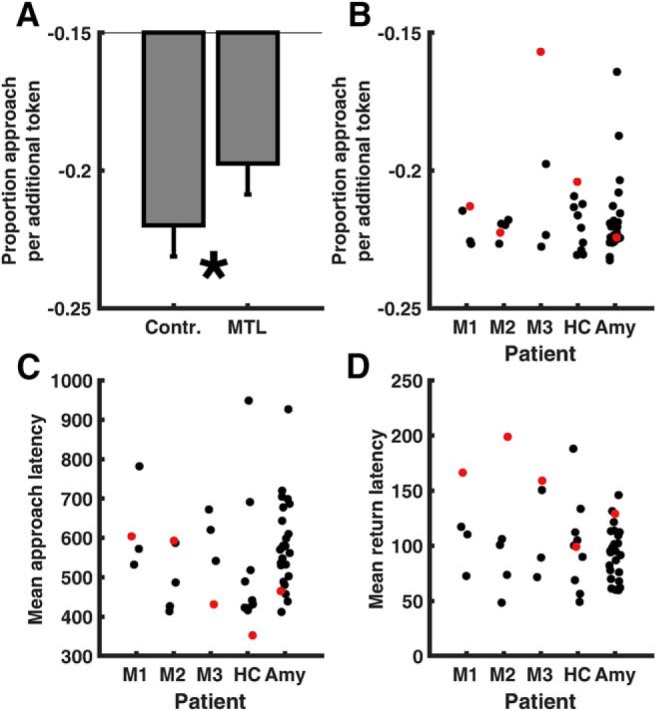
***A***, Fitted linear coefficients for the relationship between potential loss and proportion of approach, for the control and MTL lesion group (mean ± pooled SEM). ***B***–***D***, Fitted linear coefficients (***B***), mean approach latency (***C***), and mean return latency (***D***), for individual MTL patients (M1–M3), selective bilateral hippocampus lesion patient (HC), and selective bilateral amygdala lesion patient (Amy). Red dots indicate patients. *p < 0.05, one-tailed.

Next, we tested our hypothesis that patients with MTL lesions would be more likely than control participants to approach as potential loss increased. As expected, the linear relation of potential loss with the proportion of approach differed between MTL lesions and control individuals (Fig. 2*B*; Table 3). This result was confirmed in a *post hoc* test of fitted linear coefficients (one-tailed, *t*_(11)_ = 1.98, *p* = 0.037), which are depicted in Figure 4*A*. The difference between patients with MTL lesions and controls was not better explained by accounting for overall estimate of catch probability, years of education, or visual memory (Rey–Osterrieth complex figure test).

**Table 3.** Parametric comparison of MTL lesion patients (*n* = 3) and control participants (*n* = 10)

	Action (proportion approach)				Approach latency			Return latency		
	*F*	df	epsilon	*p*	*F*	df	*p*	*F*	df	*p*
Group	1	1,187		0.319	2.25	1,12,577.6	0.133	19.04	1,10,061.1	<0.001*
Group × threat level	3.5	2,187	0.9762	0.033*	1.32	2,12,577.6	0.267	0.21	2,10,061.1	0.815
Group × threat level: linear	0.17	1,187		0.683	0.56	1,12,577.6	0.453	0.34	1,10,061.1	0.562
Group × potential loss	2.23	4,187	0.4751	0.135	1.19	4,12,577.6	0.313	0.47	4,10,061.1	0.755
Group × potential loss: linear	6.57	1,187		0.011*	2.16	1,12,577.6	0.142	0.29	1,10,061.1	0.589
Interaction Group × threat level × potential loss	1.49	8,187	0.2408	0.239	0.48	8,12,577.6	0.871	0.58	8,10,061.1	0.798
Interaction Group × linear × linear	5.34	1,187		0.022	0.52	1,12,577.6	0.47	0.2	1,10,061.1	0.655

Group: patients/controls. Main effects and interactions not pertaining to group differences are omitted from the table. For all outcome measures, the table shows Satterthwaite approximation to degrees of freedom. For action, *p* values are based on further Greenhouse–Geisser correction to degrees of freedom using the epsilon value shown.

*p < 0.05.

Next, we compared the impact of selective HC (Fig. 2*C*) or amygdala lesions (Fig. 2*D*) with the unselective MTL lesions, using an ordinal dissociation test. This test allows a statement whether the observed rank difference between two patients is larger than the rank difference between random pairs of control subjects. As visible in Figure 4*B*, two of three MTL patients as well as the selective HC patient had less negative linear coefficients than all their respective controls. Indeed, the impairment of the HC patient was even slightly more pronounced than the MTL lesion group (ordinal dissociation test: *p* = 0.062). In contrast, the amygdala patient did not differ from its control group and ranked at the 30th percentile, which was significantly different from the MTL patients (ordinal dissociation test: *p* = 0.010) and from the selective HC lesion patient (ordinal dissociation test: *p* = 0.001). Collectively, these results suggest that the decision to approach is impaired due to hippocampus, but not due to amygdala, lesions.

We also found an impact of MTL lesion on the relation between threat level and approach decision (Table 3). Because the MTL and control group differed in the subjective estimation of catch rate, we replicated this result in a model with subjective catch probability as a linear predictor, as opposed to categorical threat level (*F*_(1,207.9)_ = 9.18, *p* = 0.003). Further investigating this latter result, we extracted the linear coefficient of the relation between catch probability and approach rate. For this coefficient, selective HC and amygdala patients had a nonsignificantly less pronounced deficit than the MTL patients (percentage ranks: MTL 84%, HC 55%, amygdala 50%; MTL vs HC: *p* = 0.077, MTL vs amygdala: *p* = 0.057). This suggests that the deficit may be due to lesions outside HC/amygdala.

### Selective HC and amygdala but not MTL lesions may impact approach latency

We then analyzed approach latency on those trials on which participants did approach the token (Fig. 2*F*). There was no significant difference between MTL lesion patients and the control group, such that we did not plan comparisons of MTL lesion with selective lesions. Descriptively, however, HC and amygdala patients differed from their control groups in that they approached faster overall (Figs. 2*G,H,* 4*C*). Exploratory analysis revealed that the HC patient (ordinal dissociation test: *p* = 0.010) and the amygdala patient (ordinal dissociation test: *p* = 0.016) approached faster than the MTL patients. There was no significant difference between HC and amygdala patients.

### Amygdala/MTL but not selective HC lesions impact on return latency

We then analyzed return latency on those trials on which participants successfully approached without getting caught (Fig. 2*J*), where our hypothesis was a deficit in return would be not be caused by HC lesions. Across all conditions, MTL patients returned to safety more slowly than controls (Table 3). This difference between MTL patients and controls was not better explained by accounting for subjective catch rate, years of education, or visual memory. There were no other significant differences between MTL patients and controls. We then tested our hypothesis that this effect was specific to amygdala lesions, and compared MTL patients with selective HC and amygdala lesions (Fig. 2*K*,*L*). As can be seen in Figure 4*D*, MTL patients returned more slowly than any of their control subjects, whereas in contrast, the HC patient was faster than 56% of control subjects (ordinal dissociation test: *p* = 0.024). Thus, it appears that the observed deficit in return to safety is specific to extensive MTL lesions and does not occur in selective HC lesions. In contrast, amygdala lesion patient was slower than 90% of the control group and was not significantly different from the MTL patients. Taking together all patients with unspecific MTL or amygdala lesions, they dissociated from selective HC lesion patient (ordinal dissociation test: *p* < 0.011). This suggests that the selective amygdala lesion patient was impaired in return to safety, just like MTL lesion patients, but different from the selective HC lesion patient.

### MTL lesions impact response generation but not on subjective task structure

**Figure 5.**
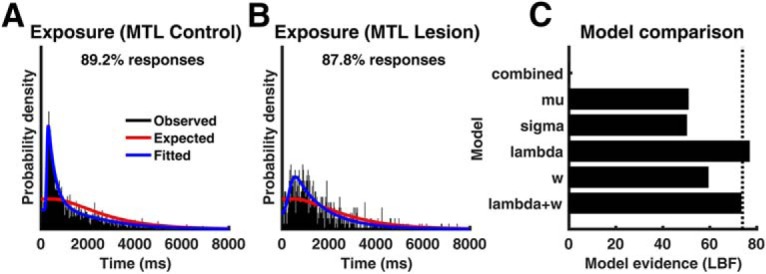
Exposure times from the safe-predator-exposure task for MTL lesion controls (***A***) and MTL lesion patients (***B***), with respect to the most recently appearing token. %Responses, Percentage of responses included in the plot; remaining responses were made before the first token occurred. Red lines show the expected exposure times under a uniform null distribution across the trial. ***C***, Evidence for different models to distinguish controls and MTL patients expressed as LBF (larger is better) with respect to a reference model with no difference between the group (combined). Dashed line indicates decisiveness threshold (LBF difference >3).

Finally, we sought to disambiguate possible causes for behavioral alterations in MTL/HC patients. We have previously demonstrated in this task that healthy people behave consistent with a subjective prior assumption that the occurrence of tokens alerts the predator. They were asked in a separate part of the game to indicate when they thought the predator was awake, and instructed they would be rewarded for exposing the predator when it was indeed awake. Healthy participants predominantly guessed that the predator was awake immediately after an (irrelevant) token appeared on the screen, despite explicit instructions that tokens were irrelevant to the task, despite feedback that such relation did not exist in the task and although this behavior made the experiment last longer. Crucially, while token collection behavior changes after negative consequences, this was hardly the case for predator exposure behavior in our previous report (Bach, 2017). The same pattern was observed in the current control group (Fig. 5*A*; KS test, *p* < 0.001). The distribution of exposure times differed between each MTL or HC lesion patients and their respective control subjects (KS test, *p* < 0.001). However, there was no consistent difference between controls and MTL lesion patients regarding the mean exposure time in a *t* test. We then fit a previously validated model to the distribution of exposure times, in which exposure times are a weighted sum of a process that distributes responses evenly across a trial, and a second process that implements a simple response to the token and is modeled by an exGauss distribution. We fit a model with the same parameters for patients and controls (combined model; Fig. 5*C*), as well as several models that split up either the exGauss parameters mu or lambda, or the weighting parameter *w*, or lambda and *w*, between patients and controls. For the group of MTL patients and their respective control participants, the best fit was achieved when splitting up the parameter lambda and not the other parameters (Fig. 5*C*; LBF difference between best and second best model: 3.6). This parameter governs the decay of the simple response process and is unrelated to prior assumptions about token–predator correlations. The same winning model but with a less decisive LBF was found for the selective HC patient (LBF difference between best and second best model: 2.4). Thus, although MTL and HC lesion patients markedly differed in their response distributions from control participants, we found no evidence that this was because of different subjective priors about the structure of the task.

## Discussion

In this paper, we sought to disentangle the contribution of hippocampus and amygdala to individual actions in a human approach–avoidance conflict test. We found that HC but not amygdala lesions impacted the decision to approach reward under threat when potential loss was high. In contrast, in amygdala but not HC lesions we observed reduced return vigor after approaching threat. Additionally, unspecific MTL lesion patients but not specific HC or amygdala lesion patients were impaired in adjusting their approach rate to threat level. As an exploratory result, an alteration in behavioral inhibition was only observed in the selective HC and amygdala lesion patients. We note that this was not a planned analysis. Finally, we did not find evidence that HC lesions alter the subjective representation of threat–reward correlations, something that we have proposed to underlie approach delay in healthy individuals (Bach, 2015, 2017).

Regarding the impact of HC lesions, our current finding is in keeping with a previous result in a spatially extended human approach–avoidance conflict task. Here, patients with degenerative HC lesions were more often outside a safe place when potential loss was high (Bach et al., 2014). However, in this setup it was difficult to separate threat approach from other actions including return to safety. Our current findings suggest that the impairment previously observed in HC lesion patients is due to increased threat approach under conflict. At the same time, amygdala lesion patient B.G. who showed the same deficit as HC lesion patients in this previous task (Korn et al., 2017) was in the current task not impaired in decision to approach, but instead in return to safety, an important component of our previous task as well. This underlines the necessity to separate action components to delineate the contribution of different brain structures.

Although hippocampus is traditionally investigated in the context of spatial navigation, cognitive maps, and declarative memory, another important aspect is its role in anxiety-like behavior (Calhoon and Tye, 2015). The current approach of separating action components may help to reconcile these views, by allowing a more specific inference on the type of approach–avoidance conflict behavior on which hippocampus lesions impact. For example, our current results are not predicted by a view according to which HC represents threat/reward aspects of a situation. In this case, one would have expected an alteration in approach latency and return vigor as well, because all action components in the task empirically depend on threat level and potential loss. Instead, our findings may tentatively suggest that hippocampus is specifically involved in a decision to approach under conflict, as initially suggested by Gray and McNaughton (2000). We note that because of the known role of hippocampus for learning and memory, our interpretation hinges on the assumption that lesion patients learned the task structure to the same extent as control participants. Crucially, MTL and hippocampus lesion patients showed a behavioral alteration only when token loss was high (3–5 tokens) but they behaved similarly to control participants when loss was low (0–2 tokens), i.e., showed the same reduction of approach rate with potential loss. Understanding the task structure is important at lower token loss, and it appears unlikely to observe this behavior if patients had not learned the task structure.

Previous nonhuman primate work pitting food reward against innate threat stimuli (rubber snakes) has revealed increased approach behavior after HC lesions, in line with the current study. Different from our study, however, they also observed reduced defensive behavior after HC lesions (Chudasama et al., 2008, 2009). In their test, the conflict situation lasted for 30 s and there was no incentive to act rapidly. To reconcile this with our findings, it is possible that in addition to approach decisions, HC is also involved in certain types of defensive behaviors, but excluding the rapid withdrawal behavior required in our study. As a crucial factor for involvement of HC as well as for the type of behavior elicited, defensive distance has been suggested (Fanselow and Lester, 1988; Gray and McNaughton, 2000; Blanchard et al., 2011). In contrast, the reduction in approach latency that our exploratory analysis suggested in both types of selective lesions may not be specific to approach–avoidance conflict: similar reduction in response times after HC lesions has also been observed in purely reward-related rodent tasks (Schwarting and Busse, 2017).

Impairment in return vigor after amygdala lesions is consistent with a rodent literature investigating non-conflict active avoidance (LeDoux et al., 2017; Terburg et al., 2018). Return to safety is a crucial component of many ethological approach–avoidance conflict tests, including our previous human version. It remains to be shown how this relates to the inconsistent reports of an amygdala role in various approach–avoidance conflict tests (Kirlic et al., 2017). Furthermore, recent work has highlighted how subregions within amygdala regulate approach toward, or avoidance of, threat in the absence of explicit or putative reward (Miller et al., 2019).

As a side finding, only unspecific MTL lesion patients were impaired in adjusting their approach rate to threat level. Although evidence for dissociation between MTL and selective lesions was not significant, one may speculate that this deficit is due to lesions outside HC or amygdala. Furthermore, different from the other findings, it may be explained by impairment in learning color-threat level association sufficiently.

As a limitation, our human lesion approach is agnostic to the contribution of HC and amygdala subregions, or microcircuitry on the level of transmitter systems. For example, anterior CA1 and CA3 appear to intricately balance their contributions to approach–avoidance conflict behavior such that specific lesions have opposing effects (Schumacher et al., 2018). Also, lesions specific to the serotonergic system within amygdala may have effects that depend on the type of conflict test (Sommer et al., 2001). For another example, anterior hippocampus inactivation had no impact on approach behavior in a specific approach–avoidance conflict task in marmosets, while increasing glutamine levels did (Wallis et al., 2019). Furthermore, a growing body of evidence suggests a longitudinal axis specialization of the hippocampus, with dorsal parts contributing more to spatial navigation and memory, and ventral parts more to anxiety tests (Strange et al., 2014). There appears to be no clear distinction between different hippocampal regions, but rather a gradient of functional contribution to different tasks. Such subtle distinctions cannot be made in clinical lesion model used here. Nevertheless, by investigating more and diverse lesion types within MTL, it may be possible to ultimately triangulate the specific function of anatomical or functional subdivisions. Furthermore, back-translating our approach of separating action components to rodents (Oberrauch et al., 2019) and nonhuman primates may help to provide a clearer picture of cross-species differences.

Ultimately, finer conceptual granularity may also help translate results from approach–avoidance conflict tests into clinical questions. Indeed, decades of research on these tests have had relatively little impact on etiological concepts or treatment of anxiety disorders (Stephan et al., 2016). It appears that different action components in approach–avoidance conflict tests resemble symptoms of different disorders. In this context, we note that an important behavioral component that was not investigated in the current setup is the duration of a decision, which may be relevant to rumination and worry in generalized anxiety disorder (Craske et al., 2017), for which approach–avoidance tests are often seen as a preclinical model (Calhoon and Tye, 2015). This can be investigated in tests that extend approach–avoidance conflict over time with no incentive to act quickly, such that approach latency has a different meaning than in our task (Chudasama et al., 2008, 2009).

We have previously demonstrated a linear relation of threat level with HC gamma oscillations (Khemka et al., 2017) and, biophysically related, BOLD signal (Bach et al., 2014; Korn and Bach, 2019) in several different human approach–avoidance tasks. In the current task, we did not observe a specifically HC-lesion-induced change in the linear relation of threat level with behavior. Future work will investigate how these neuroimaging findings can be reconciled with the pattern of lesion impairment.

To summarize, our findings add to the growing evidence that implicates the human hippocampal formation in a surprising variety of non-mnemonic behaviors such as decision-making, creativity, and prospective planning. It will be important to scrutinize how the observed deficits translate into real-world behavior in clinical populations.

## References

[B1] Amemori K, Amemori S, Graybiel AM (2015) Motivation and affective judgments differentially recruit neurons in the primate dorsolateral prefrontal and anterior cingulate cortex. J Neurosci 35:1939–1953. 10.1523/JNEUROSCI.1731-14.2015 25653353 PMC4315829

[B2] Bach DR (2015) Anxiety-like behavioural inhibition is normative under environmental threat-reward correlations. PLoS Comput Biol 11:e1004646. 10.1371/journal.pcbi.1004646 26650585 PMC4674090

[B3] Bach DR (2017) The cognitive architecture of anxiety-like behavioral inhibition. J Exp Psychol Hum Percept Perform 43:18–29. 10.1037/xhp0000282 27797550 PMC5178866

[B4] Bach DR, Talmi D, Hurlemann R, Patin A, Dolan RJ (2011) Automatic relevance detection in the absence of a functional amygdala. Neuropsychologia 49:1302–1305. 10.1016/j.neuropsychologia.2011.02.032 21345345 PMC3083511

[B5] Bach DR, Hurlemann R, Dolan RJ (2013) Unimpaired discrimination of fearful prosody after amygdala lesion. Neuropsychologia 51:2070–2074. 10.1016/j.neuropsychologia.2013.07.005 23871880 PMC3819998

[B6] Bach DR, Guitart-Masip M, Packard PA, Miró J, Falip M, Fuentemilla L, Dolan RJ (2014) Human hippocampus arbitrates approach-avoidance conflict. Curr Biol 24:541–547. 10.1016/j.cub.2014.01.046 24560572 PMC3969259

[B7] Bach DR, Hurlemann R, Dolan RJ (2015) Impaired threat prioritisation after selective bilateral amygdala lesions. Cortex 63:206–213. 10.1016/j.cortex.2014.08.017 25282058 PMC4317193

[B8] Bach DR, Korn CW, Vunder J, Bantel A (2018) Effect of valproate and pregabalin on human anxiety-like behaviour in a randomised controlled trial. Transl Psychiatry 8:157. 10.1038/s41398-018-0206-7 30115911 PMC6095858

[B9] Bannerman DM, Sprengel R, Sanderson DJ, McHugh SB, Rawlins JN, Monyer H, Seeburg PH (2014) Hippocampal synaptic plasticity, spatial memory and anxiety. Nat Rev Neurosci 15:181–192. 10.1038/nrn3677 24552786

[B10] Becker B, Mihov Y, Scheele D, Kendrick KM, Feinstein JS, Matusch A, Aydin M, Reich H, Urbach H, Oros-Peusquens AM, Shah NJ, Kunz WS, Schlaepfer TE, Zilles K, Maier W, Hurlemann R (2012) Fear processing and social networking in the absence of a functional amygdala. Biol Psychiatry 72:70–77. 10.1016/j.biopsych.2011.11.024 22218285

[B11] Blanchard DC, Griebel G, Pobbe R, Blanchard RJ (2011) Risk assessment as an evolved threat detection and analysis process. Neurosci Biobehav Rev 35:991–998. 10.1016/j.neubiorev.2010.10.016 21056591

[B12] Braun M, Finke C, Ostendorf F, Lehmann TN, Hoffmann KT, Ploner CJ (2008) Reorganization of associative memory in humans with long-standing hippocampal damage. Brain 131:2742–2750. 10.1093/brain/awn191 18757465

[B13] Britton DR, Britton KT (1981) A sensitive open-field measure of anxiolytic drug activity. Pharmacol Biochem Behav 15:577–582. 10.1016/0091-3057(81)90212-4 6117083

[B14] Burnham KP, Anderson DR (2004) Multimodel inference: understanding AIC and BIC in model selection. Sociol Method Res 33:261–304. 10.1177/0049124104268644

[B15] Calhoon GG, Tye KM (2015) Resolving the neural circuits of anxiety. Nat Neurosci 18:1394–1404. 10.1038/nn.4101 26404714 PMC7575249

[B16] Chudasama Y, Wright KS, Murray EA (2008) Hippocampal lesions in rhesus monkeys disrupt emotional responses but not reinforcer devaluation effects. Biol Psychiatry 63:1084–1091. 10.1016/j.biopsych.2007.11.012 18191111

[B17] Chudasama Y, Izquierdo A, Murray EA (2009) Distinct contributions of the amygdala and hippocampus to fear expression. Eur J Neurosci 30:2327–2337. 10.1111/j.1460-9568.2009.07012.x 20092575 PMC2852103

[B18] Craske MG, Stein MB, Eley TC, Milad MR, Holmes A, Rapee RM, Wittchen HU (2017) Anxiety disorders. Nat Rev Dis Primers 3:17024. 10.1038/nrdp.2017.24 28470168 PMC11009418

[B19] Crawford JR, Garthwaite PH (2005a) Testing for suspected impairments and dissociations in single-case studies in neuropsychology: evaluation of alternatives using monte carlo simulations and revised tests for dissociations. Neuropsychology 19:318–331. 10.1037/0894-4105.19.3.318 15910118

[B20] Crawford JR, Garthwaite PH (2005b) Evaluation of criteria for classical dissociations in single-case studies by Monte Carlo simulation. Neuropsychology 19:664–678. 10.1037/0894-4105.19.5.664 16187885

[B21] Crawford JR, Howell DC, Garthwaite PH (1998) Payne and Jones revisited: estimating the abnormality of test score differences using a modified paired samples *t* test. J Clin Exp Neuropsychol 20:898–905. 10.1076/jcen.20.6.898.1112 10484700

[B22] Crawford JR, Garthwaite PH, Gray CD (2003) Wanted: fully operational definitions of dissociations in single-case studies. Cortex 39:357–370. 10.1016/S0010-9452(08)70117-5 12784893

[B23] Esfahani-Bayerl N, Finke C, Braun M, Düzel E, Heekeren HR, Holtkamp M, Hasper D, Storm C, Ploner CJ (2016) Visuo-spatial memory deficits following medial temporal lobe damage: a comparison of three patient groups. Neuropsychologia 81:168–179. 10.1016/j.neuropsychologia.2015.12.024 26765639

[B24] Esfahani-Bayerl N, Finke C, Kopp U, Moon DU, Ploner CJ (2019) Musical memory and hippocampus revisited: evidence from a musical layperson with highly selective hippocampal damage. Cortex, in press. 10.1016/j.cortex.2018.12.023 30795831

[B25] Evans DA, Stempel AV, Vale R, Ruehle S, Lefler Y, Branco T (2018) A synaptic threshold mechanism for computing escape decisions. Nature 558:590–594. 10.1038/s41586-018-0244-6 29925954 PMC6235113

[B26] Fanselow MS, Lester LS (1988) A functional behavioristic approach to aversively motivated behavior: predatory imminence as a determinant of the topography of defensive behavior. In: Evolution and learning (Bolles RC, Beecher MD, eds), pp 185–212. Hillsdale, NJ: Lawrence Erlbaum.

[B27] Finke C, Braun M, Ostendorf F, Lehmann TN, Hoffmann KT, Kopp U, Ploner CJ (2008) The human hippocampal formation mediates short-term memory of colour-location associations. Neuropsychologia 46:614–623. 10.1016/j.neuropsychologia.2007.10.004 18023459

[B28] Geller I, Seifter J (1960) A conflict procedure for the evaluation of drugs. Fed Proc 19:2.

[B29] Gray JA, McNaughton N (2000) The neuropsychology of anxiety: an enquiry into the functions of the septohippocampal system. Oxford, UK: Oxford UP.

[B30] Hurlemann R, Wagner M, Hawellek B, Reich H, Pieperhoff P, Amunts K, Oros-Peusquens AM, Shah NJ, Maier W, Dolan RJ (2007) Amygdala control of emotion-induced forgetting and remembering: evidence from Urbach–Wiethe disease. Neuropsychologia 45:877–884. 10.1016/j.neuropsychologia.2006.08.027 17027866

[B31] Ito R, Lee ACH (2016) The role of the hippocampus in approach-avoidance conflict decision-making: evidence from rodent and human studies. Behav Brain Res 313:345–357. 10.1016/j.bbr.2016.07.039 27457133

[B32] Khemka S, Barnes G, Dolan RJ, Bach DR (2017) Dissecting the function of hippocampal oscillations in a human anxiety model. J Neurosci 37:6869–6876. 10.1523/JNEUROSCI.1834-16.2017 28626018 PMC5518417

[B33] Kirlic N, Young J, Aupperle RL (2017) Animal to human translational paradigms relevant for approach avoidance conflict decision making. Behav Res Ther 96:14–29. 10.1016/j.brat.2017.04.010 28495358 PMC5548639

[B34] Korn CW, Bach DR (2019) Minimizing threat via heuristic and optimal policies recruits hippocampus and medial prefrontal cortex. Nat Hum Behav 3:733–745. 10.1038/s41562-019-0603-9 31110338 PMC6629544

[B35] Korn CW, Vunder J, Miró J, Fuentemilla L, Hurlemann R, Bach DR (2017) Amygdala lesions reduce anxiety-like behavior in a human benzodiazepine-sensitive approach-avoidance conflict test. Biol Psychiatry 82:522–531. 10.1016/j.biopsych.2017.01.018 28364943 PMC5598543

[B36] LeDoux JE, Moscarello J, Sears R, Campese V (2017) The birth, death and resurrection of avoidance: a reconceptualization of a troubled paradigm. Mol Psychiatry 22:24–36. 10.1038/mp.2016.166 27752080 PMC5173426

[B37] Luke SG (2017) Evaluating significance in linear mixed-effects models in R. Behav Res Methods 49:1494–1502. 10.3758/s13428-016-0809-y 27620283

[B38] Miller SM, Marcotulli D, Shen A, Zweifel LS (2019) Divergent medial amygdala projections regulate approach-avoidance conflict behavior. Nat Neurosci 22:565–575. 10.1038/s41593-019-0337-z 30804529 PMC6446555

[B39] Montgomery KC (1955) The relation between fear induced by novel stimulation and exploratory behavior. J Comp Physiol Psychol 48:254–260. 10.1037/h0043788 13252152

[B40] Oberrauch S, Sigrist H, Sautter E, Gerster S, Bach DR, Pryce CR (2019). Establishing operant conflict tests for the translational study of anxiety in mice. Psychopharmacology 236:2527–2541. 10.1007/s00213-019-05315-y 31286156

[B41] Pellow S, Chopin P, File SE, Briley M (1985) Validation of open- closed arm entries in an elevated plus-maze as a measure of anxiety in the rat. J Neurosci Methods 14:149–167. 10.1016/0165-0270(85)90031-7 2864480

[B42] Penny WD, Stephan KE, Mechelli A, Friston KJ (2004) Comparing dynamic causal models. Neuroimage 22:1157–1172. 10.1016/j.neuroimage.2004.03.026 15219588

[B43] Raftery AE (1995) Bayesian model selection in social research. Sociol Methodol 25:111–163. 10.2307/271063

[B44] Rempel-Clower NL, Zola SM, Squire LR, Amaral DG (1996) Three cases of enduring memory impairment after bilateral damage limited to the hippocampal formation. J Neurosci 16:5233–5255. 10.1523/JNEUROSCI.16-16-05233.1996 8756452 PMC6579309

[B45] Rodgers RJ, Cao BJ, Dalvi A, Holmes A (1997) Animal models of anxiety: an ethological perspective. Braz J Med Biol Res 30:289–304. 10.1590/S0100-879X1997000300002 9246227

[B46] Schumacher A, Villaruel FR, Ussling A, Riaz S, Lee ACH, Ito R (2018) Ventral hippocampal CA1 and CA3 differentially mediate learned approach–avoidance conflict processing. Curr Biol 28:1318–1324.e4. 10.1016/j.cub.2018.03.012 29606418

[B47] Schwarting RK, Busse S (2017) Behavioral facilitation after hippocampal lesion: a review. Behav Brain Res 317:401–414. 10.1016/j.bbr.2016.09.058 27693851

[B48] Shin MS, Park SY, Park SR, Seol SH, Kwon JS (2006) Clinical and empirical applications of the Rey-Osterrieth complex figure test. Nat Protoc 1:892–899. 10.1038/nprot.2006.115 17406322

[B49] Sommer W, Möller C, Wiklund L, Thorsell A, Rimondini R, Nissbrandt H, Heilig M (2001) Local 5,7-dihydroxytryptamine lesions of rat amygdala: release of punished drinking, unaffected plus-maze behavior and ethanol consumption. Neuropsychopharmacology 24:430–440. 10.1016/S0893-133X(00)00210-4 11182538

[B50] Stephan KE, Bach DR, Fletcher PC, Flint J, Frank MJ, Friston KJ, Heinz A, Huys QJ, Owen MJ, Binder EB, Dayan P, Johnstone EC, Meyer-Lindenberg A, Montague PR, Schnyder U, Wang XJ, Breakspear M (2016) Charting the landscape of priority problems in psychiatry, part 1: classification and diagnosis. Lancet Psychiatry 3:77–83. 10.1016/S2215-0366(15)00361-2 26573970

[B51] Strange BA, Witter MP, Lein ES, Moser EI (2014) Functional organization of the hippocampal longitudinal axis. Nat Rev Neurosci 15:655–669. 10.1038/nrn3785 25234264

[B52] Talmi D, Hurlemann R, Patin A, Dolan RJ (2010) Framing effect following bilateral amygdala lesion. Neuropsychologia 48:1823–1827. 10.1016/j.neuropsychologia.2010.03.005 20227427 PMC2877879

[B53] Terburg D, Scheggia D, Triana Del Rio R, Klumpers F, Ciobanu AC, Morgan B, Montoya ER, Bos PA, Giobellina G, van den Burg EH, de Gelder B, Stein DJ, Stoop R, van Honk J (2018) The basolateral amygdala is essential for rapid escape: a human and rodent study. Cell 175:723–735.e16. 10.1016/j.cell.2018.09.028 30340041 PMC6198024

[B54] Vogel JR, Beer B, Clody DE (1971) Simple and reliable conflict procedure for testing anti-anxiety agents. Psychopharmacologia 21:1–7. 10.1007/BF00403989 5105868

[B55] Wallis CU, Cockcroft GJ, Cardinal RN, Roberts AC, Clarke HF (2019) Hippocampal interaction with area 25, but not area 32, regulates marmoset approach-avoidance behavior. Cereb Cortex. Advance online publication. Retrieved August 15, 2019. doi:10.1093/cercor/bhz015. 30796800 PMC6917514

